# Comparing measures of hematologic response after high-dose melphalan and stem cell transplantation in AL amyloidosis

**DOI:** 10.1038/s41408-020-00354-7

**Published:** 2020-09-01

**Authors:** Shayna Sarosiek, Luke Zheng, J. Mark Sloan, Karen Quillen, Dina Brauneis, Vaishali Sanchorawala

**Affiliations:** 1 Amyloidosis Center, Boston University School of Medicine, Stem Cell Transplantation Program in the Section of Hematology and Medical Oncology, Boston Medical Center, Boston, MA USA; 2 Department of Biostatistics, Boston University School of Public Health, Boston, MA USA

**Keywords:** Haematological diseases, Myeloma

## Abstract

Hematologic complete response (hemCR) in AL amyloidosis requires absence of monoclonal protein by immunofixation electrophoreses (IFE) and normal serum free light chain ratio (FLCR). Recent literature suggests that an involved free light chain (iFLC) <20 mg/L or difference in free light chains (dFLC) <10 mg/L may more accurately predict outcomes after treatment. We evaluated overall survival in 340 patients treated with high-dose melphalan and stem cell transplantation (SCT). Of 305 patients evaluable 6 months after SCT, 90 (30%) achieved hemCR, 132 (43%) dFLC <10 mg/L, 118 (39%) iFLC <20 mg/L, and 176 (58%) normal FLCR. Of 215 patients without hemCR, 65 (30%) had dFLC <10 mg/L and 86 (40%) had normal FLCR. Overall survival (OS) in those achieving dFLC <10 mg/L or normal FLCR without hemCR was inferior to those achieving hemCR (*p* = 0.013 and *p* = 0.001). OS was not significantly different in patients achieving iFLC <20 mg/L without hemCR compared with hemCR (*p* = 0.243). Of those with hemCR, OS was not significantly improved if dFLC <10 mg/L was also achieved (*p* = 0.852), but OS was improved for those with hemCR who also attained iFLC <20 mg/L (*p* = 0.009). Multivariate analysis demonstrated absence of monoclonal protein in IFE and iFLC <20 mg/L as independent predictors of survival. Attainment of hemCR remains a treatment goal, although achieving iFLC <20 mg/L may also predict improved OS.

## Introduction

Immunoglobulin light chain (AL) amyloidosis is a life threatening hematologic disorder characterized by deposition of amyloid fibrils formed from misfolded and aggregated clonal light chains. Accumulation of amyloid fibrils leads to renal or cardiac dysfunction in the vast majority of patients. Treatment of AL amyloidosis is directed at the underlying clonal plasma or lymphoplasmacytic cells in the bone marrow that are the source of the amyloidogenic light chains. The current criteria used to evaluate hematologic response after treatment were developed in 2012 and validated in patients undergoing high-dose melphalan and stem cell transplantation (HDM/SCT)^1,2^. These criteria define a complete hematologic complete response (hemCR) as the absence of monoclonal protein in serum and urine by immunofixation electrophoresis (IFE) and a normal serum free light chain ratio (FLCR). Very good partial response (VGPR) is defined as a difference in involved and uninvolved serum free light chains (dFLC) < 40 mg/L and partial response (PR) as a decrease in the dFLC by >50% compared to baseline.

After achieving a hematologic response, ideally a hematologic CR (hemCR), a patient is typically monitored to assess for achievement of an organ response. Organ response lags behind hematologic response with renal response occurring on average 7 months after treatment initiation (range, 1–120) and cardiac response at an average of 14 months (range, 2–78)^3^. Time to maximum organ improvement may be years after effective treatment. Recent data suggest that organ responses, as well as other patient outcomes, depend on the depth of hematologic response. Manwani et al. reported that in a cohort of 915 patients treated with upfront bortezomib, the achievement of a stringent dFLC response (defined as a dFLC < 10 mg/L) resulted in improved overall survival (OS) and time to next treatment when compared with those patients that did not achieve a dFLC < 10 mg/L^4^. Additional data also demonstrate that an involved serum free light chain (iFLC) ≤ 20 mg/L predicts for better OS in patients with AL amyloidosis who have an underlying lambda plasma cell dyscrasia^5^. Similarly, achievement of a hemCR or VGPR with absence of a monoclonal protein by IFE in combination with a low/normal iFLC is reported to lead to improved progression free survival, longer OS, and higher rates of organ response^6^.

Achievement of a dFLC < 10 mg/L or an iFLC < 20 mg/L have not been incorporated into the hematologic response criteria for AL amyloidosis. Moreover, these criteria have not been evaluated in a population of patients treated only with HDM/SCT. This study was designed to evaluate OS in patients with or without a hemCR after HDM/SCT comparing those who achieved an iFLC < 20 mg/L, a dFLC < 10 mg/L, or a normal FLCR.

## Methods

All patients with AL amyloidosis treated with HDM/SCT between January 2003 and December 2018 at Boston Medical Center and Boston University Amyloidosis Center were included in this analysis of prospectively collected data. Eligibility for HDM/SCT was based on previously described criteria^7^. Informed consent for prospective data collection was obtained in accordance with the Declaration of Helsinki and the study was approved by the Boston University Medical Center Institutional Review Board. Demographic, clinical, and laboratory data were collected and analyzed for each patient. Hematologic response according to International Society of Amyloidosis validated criteria was assessed at ~6 months, the typical interval for evaluation after HDM/SCT. Based on the aforementioned criteria, bone marrow examination was not required for assessment of hematologic response. Serum FLCs were quantified with Freelite reagent sets using the Binding Site Optilite analyzer (The Binding Site Ltd) with reference intervals of 3.3–19.4 mg/L for kappa, 5.7–26.3 mg/L for lambda, and 0.26–1.65 for the FLCR. Organ involvement was based on Congo red positive staining or clinical criteria^1,8^. Renal staging was based on estimated glomerular filtration rate and proteinuria per established criteria^9^ and cardiac staging was determined by troponin-I and B-type natriuretic peptide per the Boston University cardiac staging system^10^. Kaplan–Meier survival curves were generated to compare survival between patients with hemCR by International Society of Amyloidosis criteria compared with those not achieving a CR but with an iFLC < 20 mg/L, a dFLC < 10 mg/L, or a normal FLCR. In addition, survival was compared in patients achieving a hemCR in addition to an iFLC < 20 mg/L or a dFLC < 10 mg/L. Survival in patients with an iFLC < 20 mg/L with and without a hemCR was also compared. Moreover, survival in patients with absence of monoclonal protein on IFE and achievement of either iFLC < 20 mg/L or dFLC < 10 mg/L were also compared with patients achieving a hemCR. Hazard ratios (HR) for death and 95% confidence intervals (CI) were estimated using multivariate Cox proportional hazards regression models. In order to determine which hematologic variable predicts survival, different models were constructed, and their concordance (C-Statistic) and area under the curve (AUC) were performed.

## Results

During the specified 16 years, a total of 340 patients with AL amyloidosis were treated with HDM/SCT. Thirty-five patients (10%) were excluded from analysis due to death prior to 6 month assessment after SCT (*n* = 22, 6%) or lack of follow-up (*n* = 13, 4%). Of those evaluable at 6 months (*n* = 305), the median age was 57 years and the majority of patients were male. Baseline demographics and clinical characteristics are outlined in Table 1. The hematologic responses of all evaluable patients at 6 months following HDM/SCT were as follows: 90 patients (30%) with hemCR, 135 (44%) with VGPR, 35 (12%) with PR, and 45 (15%) with no response/progression of disease. Of the evaluable patients, 132 (43%) had a dFLC < 10 mg/L, 118 (39%) had an iFLC < 20 mg/L, and 176 (58%) had a normal FLCR. Of the 215 patients without a hemCR, 63 (29%) had an iFLC < 20 mg/L, 65 (30%) had a dFLC < 10 mg/L, and 86 (40%) had a normal FLCR. Of the 90 patients that achieved a hemCR, 55 (61%) achieved an iFLC < 20 mg/L and 64 (71%) achieved a dFLC < 10 mg/L. Sixteen patients (5%) achieved a modified CR, as defined by absence of monoclonal protein on serum and urine IFE with a normal iFLC level but an abnormal FLCR. In addition, 36 patients (12%) achieved a hematologic CR, but with an iFLC that was > 20 mg/L.

**Table 1:** Baseline patient characteristics.

			Not achieving hematologic complete response (*n* = 215)^a^		
	Total cohort (*n* = 305)	Complete hematologic response (*n* = 90)	iFLC < 20 mg/L (*n* = 63)	dFLC < 10 mg/L (*n* = 65)	normal FLCR (*n* = 86)
Median age at time of SCT, range	57 (26–77)	56 (26–70)	57 (36–74)	58 (35–74)	58 (35–77)
Females, *n* (%)	115 (38)	36 (40)	34 (55)	31 (48)	31 (36)
Organ involvement					
Cardiac, *n* (%)	160 (53)	46 (51)	21 (34)	26 (41)	37 (44)
Renal, *n* (%)	240 (79)	74 (82)	49 (79)	53 (83)	78 (92)
Liver, *n* (%)	37 (12)	17 (19)	6 (10)	8 (13)	12 (14)
Peripheral nervous system, *n* (%)	32 (11)	14 (16)	4 (7)	5 (8)	4 (5)
Autonomic nervous system, *n* (%)	51 (17)	16 (18)	10 (16)	14 (22)	15 (18)
Boston University Cardiac Stage^b^					
Stage I, *n* (%)	82 (27)	22 (24)	26 (42)	26 (41)	24 (28)
Stage II, *n* (%)	68 (22)	19 (21)	11 (18)	11 (17)	15 (18)
Stage III, *n* (%)	26 (9)	7 (8)	5 (8)	5 (8)	8 (9)
Stage IIIb, *n* (%)	8 (3)	3 (3)	0	2 (3)	3 (4)
Renal stage^c^					
Stage I, *n* (%)	143 (47)	33 (37)	29 (47)	29 (45)	30 (35)
Stage II, *n* (%)	117 (38)	37 (41)	26 (42)	27 (42)	36 (42)
Stage III, *n* (%)	42 (14)	18 (21)	8 (13)	9 (14)	18 (21)
Hematologic parameters					
Median dFLC, mg/L (range)^d^	79 (0–7094)	60 (0.3–2282)	28 (0.1–879)	28 (0.1–771)	36 (0.1–1157)
Bone marrow plasma cell % (range)	10 (0–50)	10 (0–50)	10 (0–30)	10 (0–30)	10 (0–30)
Melphalan dose					
100 mg/m^2^, *n* (%)	13 (4)	2 (2)	2 (3)	4 (6)	7 (8)
140 mg/m^2^, *n* (%)	103 (34)	27 (30)	17 (27)	21 (33)	32 (37)
200 mg/m^2^, *n* (%)	189 (62)	61 (68)	44 (71)	40 (63)	47 (55)

*SCT* stem cell transplantation, *dFLC* difference between involved and uninvolved free light chains.

^a^Of those without a hematologic complete response, patients may be included in 0 or ≥1 category depending on free light chain results.

^b^Cardiac staging data unavailable in 121 patients of total cohort.

^c^Renal staging data unavailable in 3 patients of total cohort.

^d^dFLC calculations were performed using a value of 0 for all FLC levels reported as less than measurable in 26 patients.

**Fig. 1:**
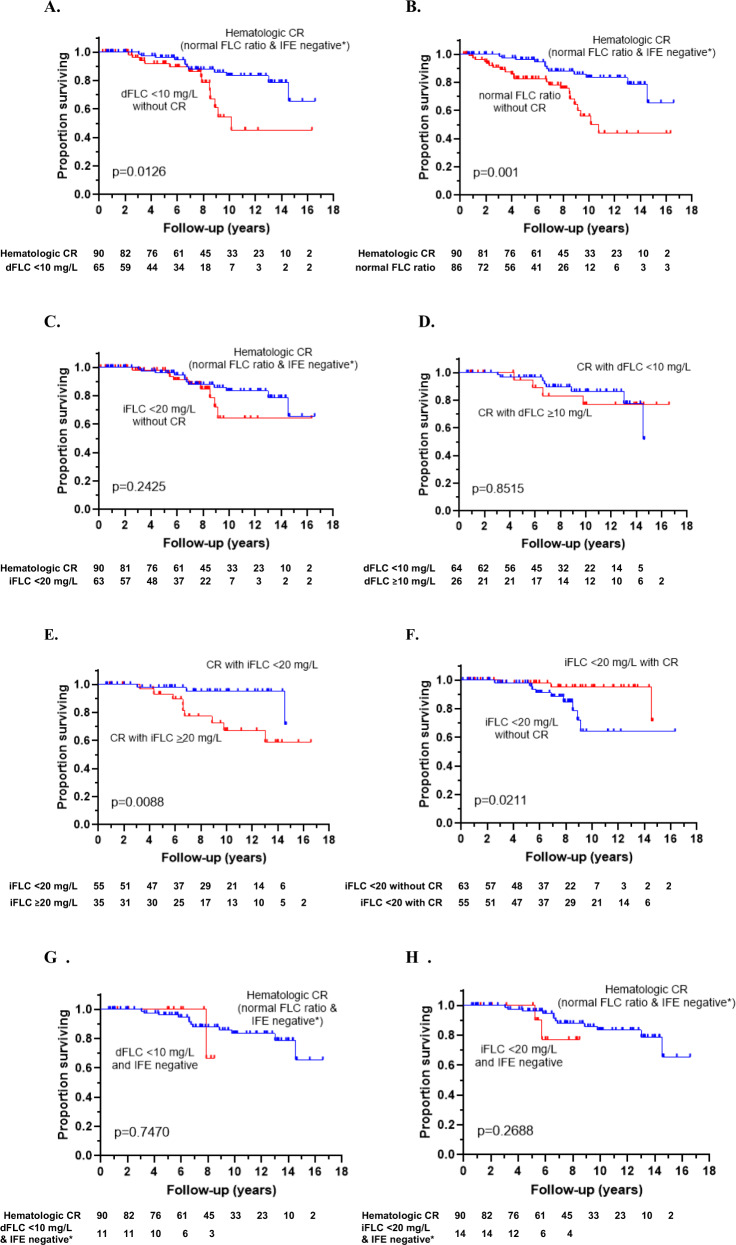
Overall survival of patients with hematologic complete response (hemCR) versus those without hemCR following treatment with HDM/SCT in AL amyloidosis. **a** Median overall survival for the patients with hemCR versus dFLC < 10 mg/L without hemCR (*p* = 0.013); not reached vs 10.2 years. **b** Median overall survival for the patients with hemCR versus normal FLCR without hemCR (*p* = 0.001); Not reached vs 10.8 years. **c** Median overall survival for the patients with hemCR versus iFLC < 20 mg/L without hemCR (*p* = 0.242); Not reached for both groups. **d** Median overall survival for the patients with hemCR with dFLC < 10 mg/L versus hemCR with dFLC ≥10 mg/L (*p* = 0.852); not reached for both groups. **e** Median overall survival for the patients with hemCR with iFLC < 20 mg/L versus hemCR with iFLC ≥ 20 mg/L (*p* = 0.008); median OS not reached for both groups. **f** Median overall survival for the patients with iFLC < 20 mg/L with or without hemCR (*p* = 0.021); not reached for both groups. **g** Median overall survival for the patients with hemCR versus dFLC < 10 mg/L and IFE negative (*p* = 0.747); not reached for both groups. **h** Median overall survival for the patients with hemCR versus iFLC < 20 mg/L and IFE negative (*p* = 0.269); not reached for both groups.

Median follow-up for evaluable patients was 7.5 years. OS was significantly longer in the group that achieved a hemCR compared to those with a dFLC < 10 mg/L without a hemCR (median OS not reached vs. 10.2 years, *p* = 0.013) (Fig. 1a). Similarly, those with a normal FLCR without a hemCR had significantly worse OS compared with those with a hemCR (median OS 10.8 years vs. not reached, *p* = 0.001) (Fig. 1b). OS was similar for patients achieving a hemCR compared to those with an iFLC < 20 mg/L without a hemCR (median OS not reached for either group, *p* = 0.241) (Fig. 1c). Of those with a hemCR, OS was not significantly improved if a dFLC < 10 mg/L was also achieved (*p* = 0.852) (Fig. 1d), but for those with a hemCR who also attained an iFLC < 20 mg/L median OS was significantly improved compared to a hemCR alone (median OS not reached for either group, *p* = 0.009) (Fig. 1e). OS at 10 years was 95% for those achieving a hemCR with an iFLC < 20 mg/L and 64% for those without a hemCR with an iFLC < 20 mg/L (median survival not reached for either group, *p* = 0.021) (Fig. 1f). There was no statistically significant difference in OS between those with hemCR and those with dFLC < 10 mg/L and absence of monoclonal protein on IFE (*p* = 0.747) (Fig. 1g). Similarly, there was no significant difference in OS between those with hemCR and those with iFLC < 20 mg/L and absence of monoclonal protein on IFE (*p* = 0.269) (Fig. 1h). Of note, the analyses for Fig. 1g, h are limited due to small sample size.

Multivariate Cox proportional hazard models demonstrate that IFE negativity (absence of monoclonal protein in serum and urine IFEs) and iFLC < 20 mg/L were independent predictors of survival. In contrast, dFLC < 10 mg/L was not predictive of survival. Although not statistically significant, our model demonstrates a trend toward improved OS in patients with normal FLCR (HR, 0.590; 95% CI 0.345–1.007; *p* = 0.053) (Table 2).

**Table 2:** Multivariate analysis predicting overall survival using different hematologic variables.

	Model 1: iFLC and dFLC C-statistic = 0.6575 AUC = 0.6824 *n* = 305		Model 2: IFE and FLCR C-statistic = 0.6973 AUC = 0.7070 *n* = 268		Model 3: IFE, iFLC and dFLC C-statistic = 0.7136 AUC = 0.7354 *n* = 305	
Variable	HR (95% CI)	*p* value	HR (95% CI)	*p* value	HR (95% CI)	*p* value
IFE negative			0.320 (0.590, 0.637)	**0.0012**	0.374 (0.202, 0.690)	**0.0017**
Normal FLCR			0.590 (0.345, 1.007)	0.0532		
iFLC < 20 mg/L	0.199 (0.093, 0.425)	**<0.0001**			0.199 (0.092, 0.434)	**<0.0001**
dFLC < 10 mg/L	1.043 (0.562, 1.934)	0.8949			1.528 (0.787, 2.967)	0.2106

*IFE* absence of monoclonal protein in serum and urine immunofixation electrophoresis, *FLCR* free light chain ratio, *iFLC* involved free light chain level, *dFLC* difference in involved and uninvolved free light chain level, *C-Statistic* concordance, *AUC* area under the curve, *HR* hazard ratio, *CI* confidence interval.

Bold denotes statistically significant value.

## Discussion

These data demonstrate that achievement of a hemCR using established criteria is superior to achieving only a dFLC < 10 mg/L or a normal FLCR for patients undergoing treatment with HDM/SCT for AL amyloidosis. This supports the idea that absence of a monoclonal protein by serum and urine immunofixation electrophoresis is an important part of the hematologic response criteria, as the majority of patients that did not meet criteria for a hemCR had continued presence of monoclonal protein by immunofixation. No significant difference was seen in OS for patients achieving an iFLC < 20 mg/L compared with a hemCR, despite 27 (44%) of those with an iFLC < 20 mg/L having an abnormal FLCR, suggesting that this parameter is likely more predictive than a normal FLCR. These findings are also supported by our multivariate analysis demonstrating independent predictive value of immunofixation electrophoresis and iFLC.

Free light chain levels can be affected by a number of clinical factors, including impaired renal function^11^, underlying inflammatory or infectious conditions^12^, and new monoclonal antibody therapies such as daratumumab and isatuximab^13^. Therefore free light chain measurements may not accurately reflect an individual’s hematologic response. Despite this fact, we found that additional improvement in OS is seen in patients with a hemCR who also achieve an iFLC < 20 mg/L. This supports the claim that a maximal FLC response (lower than the standard upper limit of normal for the lambda serum free light chain) may be associated with even better outcomes in those with a hemCR. Newer assessments of hematologic response, such as minimal residual disease measurement by next generation sequencing and multiparametric flow cytometry may be additional manners of measuring deeper hematologic responses^14,15^. Novel modalities that detect monoclonal proteins with higher sensitivity, such as next generation sequencing, multiparametric flow cytometry, matrix-assisted laser desorption ionization time-of-flight, and mass-spectrometry-based assay may also prove to predict patient outcomes^16,17^.

A recent report from the UK National Amyloidosis Center reported that the achievement of dFLC < 10 mg/L in a cohort of 915 patients treated with upfront bortezomib resulted in improved OS and time to next treatment when compared with those patients that did not achieve a dFLC < 10 mg/L^4^. However, our study failed to show the predictive power of dFLC < 10 mg/L with respect to OS by Kaplan–Meier analysis as well as by multivariate models. This may be related to smaller sample size as well as different treatment regimens as all the patients in our study were treated with HDM/SCT.

This study is limited by its retrospective nature. These findings require additional validation in larger prospective patient cohorts, including patients receiving non-SCT treatment modalities and those with comorbid medical conditions, such as renal failure, which may affect FLC levels. Despite these limitations, our data support the conclusion of recent publications indicating improvement in outcomes in patients with a hemCR (with absent monoclonal protein by immunofixation electrophoresis) and low serum free light chain levels.

In conclusion, achievement of a hemCR by the established hematologic response criteria (which includes the absence of monoclonal protein on IFE) is superior to achieving only a dFLC <10 mg/L or a normal FLCR. The potential inclusion of an iFLC <20 mg/L, or perhaps other markers of a deeper hematologic response, in addition to serum FLCR normalization may lead to better prediction of OS in patients with AL amyloidosis and should be further investigated.
